# Clinical predictors of rectal lymphogranuloma venereum infection: results from a multicentre case–control study in the UK

**DOI:** 10.1136/sextrans-2013-051401

**Published:** 2014-03-31

**Authors:** S N S Pallawela, A K Sullivan, N Macdonald, P French, J White, G Dean, A Smith, A J Winter, S Mandalia, S Alexander, C Ison, H Ward

**Affiliations:** 1 Chelsea and Westminster Hospital NHS Foundation Trust, London, UK; 2 Department of Infectious Disease Epidemiology, School of Public Health, Imperial College London, London, UK; 3 Mortimer Market Centre, Central and North West London NHS Foundation Trust, London, UK; 4 Guy's and St Thomas’ NHS Foundation Trust, London, UK; 5 Brighton & Sussex University Hospitals NHS Trust, Claude Nicol Centre, Brighton, UK; 6 Jefferiss Wing Centre for Sexual Health, Imperial College Healthcare NHS Trust, London, UK; 7 Sandyford Sexual Health Services, Glasgow, UK; 8 Sexually Transmitted Bacterial Reference Laboratory, Public Health England, London, UK

**Keywords:** LYMPHOGRANULOMA VENEREUM, MSM, PROCTITIS, SEXUALLY TRANSMITTED INFECTION, CLINICAL PREDICTORS

## Abstract

**Objective:**

Since 2003, over 2000 cases of lymphogranuloma venereum (LGV) have been diagnosed in the UK in men who have sex with men (MSM). Most cases present with proctitis, but there are limited data on how to differentiate clinically between LGV and other pathology. We analysed the clinical presentations of rectal LGV in MSM to identify clinical characteristics predictive of LGV proctitis and produced a clinical prediction model.

**Design:**

A prospective multicentre case–control study was conducted at six UK hospitals from 2008 to 2010. Cases of rectal LGV were compared with controls with rectal symptoms but without LGV.

**Methods:**

Data from 98 LGV cases and 81 controls were collected from patients and clinicians using computer-assisted self-interviews and clinical report forms. Univariate and multivariate logistic regression was used to compare symptoms and signs. Clinical prediction models for LGV were compared using receiver operating curves.

**Results:**

Tenesmus, constipation, anal discharge and weight loss were significantly more common in cases than controls. In multivariate analysis, tenesmus and constipation alone were suggestive of LGV (OR 2.98, 95% CI 0.99 to 8.98 and 2.87, 95% CI 1.01 to 8.15, respectively) and that tenesmus alone or in combination with constipation was a significant predictor of LGV (OR 6.97, 95% CI 2.71 to 17.92). The best clinical prediction was having one or more of tenesmus, constipation and exudate on proctoscopy, with a sensitivity of 77% and specificity of 65%.

**Conclusions:**

This study indicates that tenesmus alone or in combination with constipation makes a diagnosis of LGV in MSM presenting with rectal symptoms more likely.

## Introduction

### Background

Since 2003, an outbreak of lymphogranuloma venereum (LGV) has been recognised in Western Europe affecting men who have sex with men (MSM)^1^ with the serovar L2b identified as the causative organism.^2^ By June 2012, 2138 LGV cases had been diagnosed in the UK with 99% in MSM, the majority with established HIV infection.^3^

In this current outbreak, LGV usually presents as a primary proctitis, sometimes associated with constitutional symptoms.^4^ Anal discharge and proctitis are commonly reported,^5^ ^6^ but there are limited data on the specificity of these symptoms and signs, making it difficult to differentiate clinically between LGV and other infections. LGV proctitis may also resemble inflammatory bowel disease in symptoms, endoscopic findings and histology and may result in referral to other specialties resulting in delayed diagnosis and increased risk of complications.^7–11^ Relatively few MSM diagnosed with LGV have presented with the inguinal syndrome^12^ or perianal ulceration,^13^ and in the UK no significant reservoir of asymptomatic or undiagnosed infection has been identified, in contrast to data from the Netherlands.^14–16^ The persistence of LGV in the UK over nearly a decade shows that control efforts have failed to limit ongoing transmission.

### Objectives

We aim to describe in detail the clinical presentation and course of LGV in MSM, and to identify clinical symptoms and signs predictive of LGV compared with other forms of proctitis and to produce a clinical prediction model to support the diagnostic process.

## Methods

### Study design

A prospective multicentre case–control study. This is part of a wider study (LGV-net) that explored the clinical, epidemiological and microbiological characteristics of LGV.^17^ In the study of risk factors for LGV acquisition, two control groups were used, symptomatic and asymptomatic. However, in the analysis presented here, we are using only the symptomatic controls as we aim to identify the specific symptoms and clinical characteristics distinguishing LGV from other presentations.

### Setting

Subjects were recruited from Genitourinary Medicine, HIV and Sexual Health Clinics, based in six hospitals located in London, Brighton and Glasgow, selected to include those seeing significant numbers of cases of LGV in the UK. National ethics committee approval was granted for the study (07/H0712/156) and individual informed patient consent obtained.

### Participants

Cases were MSM diagnosed with rectal LGV (included rectal asymptomatic and presymptomatic patients) between August 2008 and December 2010 at one of the participating clinics. Controls were selected (one control per case) by staff involved in the study, from MSM presenting to the same centre during the same week as the case. The inclusion criteria for controls were patients presenting with symptoms of proctitis, anogenital ulceration or inguinal lymphadenopathy who tested negative for LGV and who had reported anogenital sex with a man in the previous 3 months. We aimed to recruit one symptomatic control for each case from the same centre, and seen in the same date period as the case. All controls reported symptoms of proctitis, some also had perianal ulceration. Ten patients initially recruited as symptomatic controls and nine asymptomatic controls who were subsequently diagnosed with LGV were transferred to the LGV case group and further controls recruited. Men who were unable to provide informed consent, who lacked sufficient English or were unable to complete the computer-assisted self-interviews (CASIs) were not eligible.

### Investigations

Cases and controls were investigated using standard clinic protocols, including testing for chlamydia and gonorrhoea from rectum, urethra/urine and pharynx by a variety of NAATs. All samples from cases and controls that tested positive for *Chlamydia trachomatis* were sent to the relevant Sexually Transmitted Bacterial Reference Laboratory (STBRL) in England or Scotland for LGV testing using an LGV-specific real-time PCR assay.^18^ Proctoscopy was carried out on all those with rectal symptoms unless there were clinical contraindications, such as significant pain or the patient declined. Serological testing for HIV, syphilis and hepatitis C was undertaken according to clinical need.

### Data collection and handling

Patients completed a web-based CASI providing detailed accounts of sociodemographics, risk behaviour and previous medical history. A separate web-based clinical report form (CRF) was completed by the recruiting team, detailing patient symptoms, relevant past medical and sexual history, examination findings and results of investigations. Patients were offered a full sexual health screen and received standard clinical care. Cases were followed up by undergoing a test of cure 4 weeks and 6 months after completion of treatment and re-screened for all sexually transmitted infections (STIs) including HIV, syphilis and hepatitis C if initially negative. Cases were asked to complete a further CASI at the test of cure follow-up concerning experiences of LGV symptoms and treatment.

Data from the online CASI and the CRF were linked using a unique study number; no personal identifying information was collected. Data were then combined into a standard database format, cleaned and exported to a statistical package for analysis.

### Main outcome measure and statistical analysis

A preliminary descriptive univariate analysis was carried out comparing the clinical presentation and findings for cases and controls. Continuous variables were initially explored using t tests. Missing data were omitted. Key symptoms and signs (p<0.05) predictive of LGV were then analysed further in a multivariable logic regression model. The final multivariable model presented shows significant independent predictors of LGV adjusted for the residual or confounding effects of other variables in the model. Using the results of the univariate and multivariate analysis, we constructed alternative clinical prediction models for LGV in men with rectal symptoms. Symptoms and signs with the largest ORs were entered into different models and receiver operating curves plotted of 1-specificity against sensitivity to identify an algorithm to support the diagnosis of LGV.

## Results

### Participants

In total, 98 rectal LGV cases and 81 symptomatic controls between August 2008 and December 2010. The recruitment rate was 84%; 78% for cases and 87% controls.

### Descriptive data

Both cases and controls were mainly men in their 30s and 40s, around half of whom were born in the UK, and the majority of whom reported previous STIs (table 1). Cases were significantly more likely than controls to be of white ethnicity (90% vs 78%), to be coinfected with HIV (90% vs 72%) and to have had one or more previous STIs (99% vs 84%). Controls had a wide range of eventual diagnoses: 44 had one or more STIs (table 2), several patients had coinfections, 30 had no specific diagnosis and 7 had non-STI diagnoses (3 haemorrhoids, 2 anal fissures, 1 giardia and 1 microsporidiosis). Ten (10%) LGV cases and eight (10%) symptomatic controls gave a history of hepatitis C. Six LGV cases had a recently (within 6 months) positive hepatitis C PCR with no previous history of hepatitis C detected at the follow-up visit for LGV. Among the nine LGV cases known not to have HIV infection at baseline, one seroconverted for HIV within the 6-month follow-up.

**Table 1:** Clinic attendance, demographic information and history of sexually transmitted infections for rectal LGV cases compared with symptomatic controls

Characteristic	Cases N (%)	Controls N (%)	OR (95% CI)	p Value
** **	N=98	N=81		
Recruitment setting				
HIV clinic	65 (66)	55 (68)	Ref	0.757*
Genitourinary medicine clinic	27 (28)	23 (28)	0.99 (0.51 to 1.93)	
Other†	6 (6)	3 (4)	1.69 (0.40 to 7.08)	
Reason attended clinic				
Symptoms	75 (78)	72 (89)	Ref	0.075*
Routine/check up	12 (12)	3 (4)	3.84 (1.04 to 14.17)	
Other‡	9 (9)	6 (7)	1.44 (0.49 to 4.25)	
Age median (IQR)	39.5 (34–46)	37 (30–44)	n/a	0.087§
Ethnicity—white (UK or other)	87 (90)	63 (79)	2.35 (1.01 to 5.47)	0.048
Born in UK	54 (56)	53 (65)	0.66 (0.36 to 1.22)	0.186
Known HIV-positive	88 (90)	58 (72)	3.49 (1.55 to 7.87)	0.003
Previous hepatitis C	10 (10)	8 (10)	1.02 (0.38 to 2.73)	0.964
Previous gonorrhoea	67 (68)	43 (54)	1.86 (1.01 to 3.43)	0.047
Previous syphilis	47 (48)	31 (39)	1.46 (0.80 to 2.65	0.219
Previous LGV	16 (16)	8 (10)	1.76 (0.71 to 4.34)	0.223
Previous chlamydia (non-LGV)	51 (52)	30 (38)	1.81 (0.99 to 3.30)	0.054
Any previous sexually transmitted infection	97 (99)	67 (84)	18.82 (2.4 to 147.3)	0.005

HIV postexposure prophylaxis related: one case and two controls. Serological tests for syphilis follow-up: one case. Confirm new HIV: one control.

*p Value for trend.

†MSM or SW dedicated clinics.

‡Contact tracing: six cases and three controls.

§Wilcoxon rank sum test for medians.

LGV, lymphogranuloma venereum; MSM, men who have sex with men; SW, sex worker.

**Table 2:** Sexually transmitted infections diagnosed in rectal LGV cases and symptomatic controls

Characteristic	Cases N (%)	Controls N (%)	OR (95% CI)	p Value
** **	N=98	N=81		
Rectal chlamydia (non-LGV)	n/a	20 (25)	n/a	
Urethral chlamydia	5 (6)	3 (4)	1.43 (0.33 to 6.20)	0.629
Pharyngeal chlamydia*	4	0	n/a	
Rectal gonorrhoea	18 (19)	6 (8)	2.81 (1.06 to 7.46)	0.038
Urethral gonorrhoea	3 (3)	2 (3)	1.28 (0.21 to 7.85)	0.791
Pharyngeal gonorrhoea	2 (2)	2 (2)	0.89 (0.12 to 6.44)	0.905
Non-gonococcal urethritis	7 (8)	2 (3)	3.09 (0.62 to 15.32)	0.168
Anogenital herpes	2 (22)	6 (8)	0.26 (0.05 to 1.34)	0.168
Anogenital warts	8 (9)	10 (13)	0.64 (0.24 to 1.71)	0.371
Syphilis (new infection)†	1 (1)	5 (7)	0.17 (0.02 to 1.46)	0.105

*20 cases and 24 controls were screened for pharyngeal chlamydia.

†78 cases and 69 controls had syphilis serology results available.

LGV, lymphogranuloma venereum.

Some cases had other STIs in addition to rectal LGV (table 2). LGV was also detected in the pharynx in one patient who presented with rectal symptoms but no pharyngeal symptoms. However, screening for pharangeal *C trachomatis* was not routine at most of the participating clinics and was carried out for only 20 cases and 24 controls included in this study.

Although the majority of the cases had rectal symptoms, nine reported no rectal symptoms at the time of their initial positive test for *C trachomatis*. Of these, four were contacts of LGV and the remainder reported other genital symptoms. One month after completion of treatment, five of the nine reported having developed at least one rectal symptom in the period between testing and treatment, two reported no symptoms throughout and two did not complete the follow-up questionnaire where LGV symptoms were recorded.

Median duration of symptoms was 13 days in cases and 7 days in controls, although eight cases and eight controls reported being symptomatic for over a month before seeking care. The most common symptoms in men with LGV were rectal discharge (66%), bleeding (61%) and anal pain (56%), followed by tenesmus (33%) and a change in bowel habit, with around one in three reporting constitutional symptoms (table 3). Tenesmus, constipation, anal discharge and weight loss were significantly more common in cases than controls. A similar proportion of cases and controls also reported genital symptoms (10% and 15%, respectively), mostly dysuria and urethral discharge.

**Table 3:** Presenting symptoms and proctoscopic findings in rectal LGV cases and symptomatic controls

Characteristic	Cases n (%)	Controls n (%)	OR (95% CI)	p Value
*Total*	98	81		
Any rectal symptoms	89 (91)	81 (100)	n/a	
Duration: median days (IQR)	13 (5–21)	7 (3.5–14.5)	n/a	0.146*
Anal discharge	65 (66)	36 (44)	2.46 (1.34 to 4.51)	0.004
Rectal bleeding	60 (61)	41 (51)	1.54 (0.85 to 2.79)	0.155
Anal pain	55 (56)	36 (44)	1.60 (0.88 to 2.89)	0.121
Tenesmus	32 (33)	6 (7)	6.06 (2.39 to 15.40)	<0.001
Constipation	29 (30)	8 (10)	3.84 (1.64 to 8.96)	0.002
Loose stools/diarrhoea	28 (29)	20 (25)	1.22 (0.63 to 2.38)	0.560
Any constitutional symptoms	33 (34)	21 (26)	1.45 (0.76 to 2.78)	0.265
Malaise	19 (20)	8 (10)	2.19 (0.90 to 5.32)	0.082
Fever	16 (16)	8 (10)	1.78 (0.72 to 4.40)	0.213
Weight loss	11 (11)	1 (1)	10.10 (1.28 to 80.06)	0.029
Any genital or inguinal symptoms	11 (11)	12 (15)	0.73 (0.30 to 1.75)	0.476
*Proctoscopy findings*†	81 (91)	73 (91)		
Normal	8 (10)	16 (22)	0.39 (0.16 to 0.97)	0.044
Proctitis	52 (65)	34 (47)	2.08 (1.08 to 3.98)	0.028
Rectal bleeding	31 (39)	13 (18)	2.87 (1.36 to 6.08)	0.006
Exudate	47 (59)	20 (28)	3.70 (1.87 to 7.32)	<0.001
Lesion or/ulcer	13 (16)	10 (14)	1.22 (0.50 to 3.00)	0.661

*Wilcoxon rank sum test for medians.

†Proctoscopy was not performed in 17 cases (9 asymptomatic, 1 unable to be performed because of pain, 2 had perianal lesions only, 1 declined, 4 reason not recorded) and 8 controls (2 because of pain, 3 perianal lesions, 1 abdominal cramps, 2 reason not recorded).

LGV, lymphogranuloma venereum.

Proctoscopy was performed on 81 (83%) cases and 73 (90%) controls (table 3) with cases significantly less likely to be reported as ‘normal’ and more likely to have proctitis, exudate and bleeding, with exudate and rectal bleeding more significantly associated with LGV on univariate analysis.

### Multivariable analyses

In a multivariate model combining these symptoms and signs (adjusting for other variables), tenesmus and constipation alone were suggestive of LGV, with an OR of 2.98 (95% CI 0.99 to 8.98) and 2.87 (95% CI 1.01 to 8.15), respectively. Constipation or tenesmus occurred in approximately half (47%) of LGV cases in our study. If tenesmus alone, or in combination with constipation, was present, this was highly suggestive that LGV was the cause of the rectal symptoms, with an OR of 6.97 (95% CI 2.71 to 17.92). These associations were modified slightly if rectal gonorrhoea was added to the model, with tenesmus becoming of borderline significance (aOR 2.82, 95% CI 0.99, 8.86), constipation becoming more strongly associated (aOR 3.45, 95% CI 1.19 to 10.04) and gonorrhoea itself being an independent predictor of LGV (aOR 4.02, 95% CI 1.17 to 13.80).

**Figure 1:**
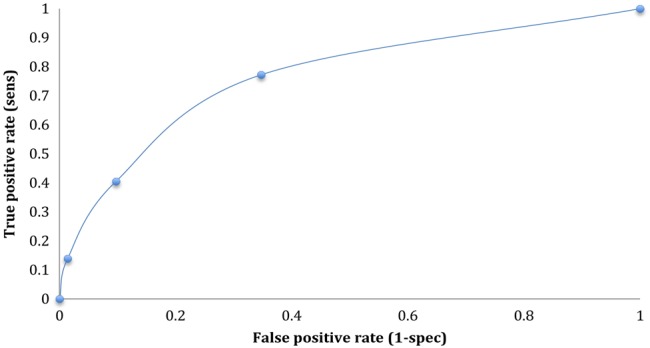
Receiver operator curve for clinical algorithm of tenesmus (1)+constipation (1)+exudate on proctoscopy (1).

We assessed potential clinical prediction models using combinations of the following symptoms and signs: tenesmus, constipation, weight loss, exudate and bleeding on proctoscopy, and a normal proctoscopy. The best balance of sensitivity and specificity was found in an algorithm combining tenesmus, constipation and exudate on proctoscopy; having at least one of these symptoms and signs gives a sensitivity of 77% and specificity of 65% (figure 1). We did not include gonorrhoea in the model since this would not be usually known at initial presentation unless suspected on Gram stain on microscopy.

The treatment regimen was recorded for 95 (97%) of the cases: the majority (90/95, 95%) received the recommended dose of doxycycline (100 mg twice daily for 3 weeks), 71 of whom had this as first line, the remainder once the diagnosis was confirmed.

## Discussion

Tenesmus, constipation, anal discharge and weight loss were significantly more common presenting symptoms in men with LGV compared with controls. In multivariate analysis, constipation and tenesmus alone were suggestive of having LGV; men with tenesmus alone or in combination with constipation were almost seven times more likely to have LGV. The median duration for rectal symptoms for LGV cases was 13 days, ranging from 1 day to a year, similar to that reported elsewhere.^19^ Proctoscopic findings helped differentiate cases and controls, with exudate or bleeding being significant predictors of LGV on univariate analysis.

We developed a simple clinical algorithm to assist in the diagnosis of LGV in men with rectal symptoms: having one or more of tenesmus, constipation and exudate on proctoscopy had a sensitivity of 77% and specificity 65%. These parameters are not high enough to be diagnostic, but may improve initial assessment, and presumptive treatment while diagnostic results are awaited. Asymptomatic rectal LGV cases were included in our analyses to reflect clinical practice; however, only 9% (9/98) of cases had no rectal symptoms at presentation: the majority were ‘presymptomatic’ and went on to develop symptoms, with only two remaining persistently asymptomatic.

This differs to the 27% asymptomatic rate observed in the Netherlands, which may reflect LGV testing of all positive rectal chlamydia specimens irrespective of symptoms (Dr Henry de Vries, personal communication). UK practice is for type-specific testing of chlamydia only in symptomatic patients and contacts.^20^

### Limitations

Our study is limited by the high number of clinicians examining patients, and therefore proctoscopic findings and rectal microscopy may not be consistently reported, and not all LGV cases had proctoscopy. Other studies have suggested that proctitis detected by proctoscopic examination together with greater than 10 or 20 poylmorphonuclear leucocytes per high-power field on a rectal smear is suggestive of LGV.^5^ ^6^

The study is strengthened by the prospective recruitment, multisite setting and systematic data collection using CASI (participants) and online CRFs (clinicians). While these methods do not eliminate variation in data quality, they will have improved the validity of reports on symptoms and signs compared with other studies of LGV that have often been based on retrospective case note review.

### Interpretation

Other studies have reported the association with tenesmus and constipation.^21–25^ Our study is the first to quantify this association that has been postulated as resulting from the transmural and perirectal inflammation and oedema caused by LGV.^25^ This study also highlights that patients with LGV present with a wide range of symptoms suggestive of other conditions and are frequently coinfected with other STIs.^7^ ^8^ ^26–29^ Nearly one-fifth of LGV cases were coinfected with rectal gonorrhoea. Most of the cases and controls were coinfected with HIV, in line with other studies in the UK,^4^ and the one case of incident HIV infection reinforces the recommendation for repeat HIV testing and offer of other risk reduction interventions. Importantly, rectal carcinoma should always be considered in those presenting with bleeding and weight loss; however, cases have been reported where LGV has presented with an ulcerating bleeding mass.^28^ ^29^

The majority of patients with LGV were treated with the recommended 3-week course of doxycycline.^20^ Due to the delay in obtaining diagnostic confirmation, treatment should be started presumptively based on clinical symptoms along with gonorrhoea treatment if clinically indicated. Patients confirmed with LGV should be offered a test of cure.

### Generalisability

These findings from a multicentre study should be applicable in areas where LGV has become established among MSM populations. The validity of the clinical predictors is highly dependent on the prevalence of LGV and the proportion of cases that are symptomatic and therefore the findings may not be applicable in settings with a different epidemic pattern for LGV.

## Conclusion

LGV is now an established infection among sexually active MSM in the UK, particularly among those with HIV. Clinicians should be aware that MSM presenting with any rectal symptoms should be tested for LGV and treated presumptively, particularly if they have one or more of tenesmus, constipation and exudate on proctoscopy.

**Key messages**

- Tenesmus alone, or in combination with constipation, is suggestive of lymphogranuloma venereum (LGV) in men who have sex with men (MSM).
- If LGV is suspected, presumptive treatment with three weeks of doxycycline should be given.
- LGV should be considered in the differential diagnosis of all MSM presenting with rectal symptoms and tests for other STIs should be carried out.
